# VitisExpDB: A database resource for grape functional genomics

**DOI:** 10.1186/1471-2229-8-23

**Published:** 2008-02-28

**Authors:** Harshavardhan Doddapaneni, Hong Lin, M Andrew Walker, Jiqiang Yao, Edwin L Civerolo

**Affiliations:** 1 USDA-ARS. San Joaquin Valley Agricultural Science Center, 9611 So. Riverbend Ave. Parlier, CA 93648, USA; 2 University of California Davis, Department of Viticulture and Enology, Davis, CA 95616, USA; 3 Citrus Research Board, 323 W. Oak, P.O. Box 230, Visalia, CA 93279, USA

## Abstract

**Background:**

The family Vitaceae consists of many different grape species that grow in a range of climatic conditions. In the past few years, several studies have generated functional genomic information on different *Vitis *species and cultivars, including the European grape vine, *Vitis vinifera*. Our goal is to develop a comprehensive web data source for Vitaceae.

**Description:**

VitisExpDB is an online MySQL-PHP driven relational database that houses annotated EST and gene expression data for *V. vinifera *and non-*vinifera *grape species and varieties. Currently, the database stores ~320,000 EST sequences derived from 8 species/hybrids, their annotation (BLAST top match) details and Gene Ontology based structured vocabulary. Putative homologs for each EST in other species and varieties along with information on their percent nucleotide identities, phylogenetic relationship and common primers can be retrieved. The database also includes information on probe sequence and annotation features of the high density 60-mer gene expression chip consisting of ~20,000 non-redundant set of ESTs. Finally, the database includes 14 processed global microarray expression profile sets. Data from 12 of these expression profile sets have been mapped onto metabolic pathways. A user-friendly web interface with multiple search indices and extensively hyperlinked result features that permit efficient data retrieval has been developed. Several online bioinformatics tools that interact with the database along with other sequence analysis tools have been added. In addition, users can submit their ESTs to the database.

**Conclusion:**

The developed database provides genomic resource to grape community for functional analysis of genes in the collection and for the grape genome annotation and gene function identification. The VitisExpDB database is available through our website .

## Background

Expressed sequence tags (ESTs) are an abundant genomic resource with 44,203,116 ESTs deposited as of July 2007 in the GenBank repository. ESTs are an important genomic resource for all species, and more so in systems for which no genome sequences are available. In such cases, ESTs are used as the basis for structural genomic annotation. ESTs are also fundamentally important for studying global expression patterns [1]. Utilization of effective bioinformatics tools has broadened the applications of EST analysis into the fields of genomics, marker development and genome annotation among others [2-4].

The *V. vinifera*-based European grapevine is the most economically important fruit species worldwide, with over 7.4 million hectares under cultivation. Grapes are produced for fruit, juice, raisins, wine and spirits. Currently, a draft genome is available, with comprehensive genome annotation still in progress [5]. Recently, several *Vitis *species genomic projects have contributed to the growing number of ESTs that are publicly available. There are numerous North American native grape species and cultivars bred from these species that have economic value and also are rich in germplasm resistant to different biotic and abiotic stresses (See Additional File 1).

Today, there are over 320,000 *Vitis *ESTs deposited in the NCBI GenBank. About 90% of these belong to *V. vinifera*, followed by 3.0% from *V. shuttleworthii*. The remainder includes ESTs from *V. arizonica*, *V. aestivalis *and *V. riparia *among others (Table 1). Of these 320,000 *Vitis *ESTs, about 200,000 ESTs derived from *V. vinifera*, *V. aestivalis *and *V. rupestris *× *V. arizonica *have been characterized [6-8]. These ESTs were derived from cDNA libraries cloned from different tissues and developmental stages during biotic and abiotic stress [6-8]. Similarly, another 5,000 ESTs cloned from Suppression Subtractive Hybridization (SSH) libraries of *V. rupestris *× *V. arizonica *hybrids in response to *Xylella fastidiosa *infection have been recently analyzed [9]. To our knowledge, there are no PUBMED references on the characterization of the full-set of available *Vitis *ESTs.

**Table 1:** Sources of *Vitis *and the ESTs sequences in the database. There are a total of 329,964 ESTs currently in the database.

**Serial #**	***Vitis *Species/Hybrids**	**Number of ESTs**	**Non-redundant ESTs**
1	*V. vinifera *(wine grape)	303,054	23148*
2	*V. shuttleworthii*	10,704	5527
3	*V. hybrid cultivar*^++^	6,533	
4	*V. rupestris × V. arizonica*^++^	5421	2928
5	*V. aestivalis*	2,101	1280
6	*V. riparia*	1,910	937
7	*V. pseudoreticulata*	122	96
8	*V. cinerea × V. rupestris*	61	53
9	*V. cinerea × V. riparia*	58	48

*Number as in the NCBI UniGene Built used in the database.

^++ ^These two EST sources that were derived from the *V. rupestris × V. Arizonica hybrids *and therefore were merged for further analysis.

In 2004, as part of its GeneChip^® ^Consortia Program, Affymetrix introduced the 16K array GeneChip^® ^*Vitis vinifera *(Grape) Genome Array ver. 1.0 (Affymetrix^® ^Inc., Santa Clara, CA), fabricated mainly for *V. vinifera *microarray studies. Recently, there have been six reports of mRNA expression profiling studies using either cDNA or oligo arrays for measuring gene expression profiles for flowers and berry skin development [10-12], and from water-deficit and iso-osmotic salinity stress in grapevine shoot tissues [13], as well as expression profiles associated with viral diseases [14] and tissue specific profiles on berry tissues [15]. GrapePLEX is a MIAME-compliant and Plant Ontology-enhanced expression database for *Vitis *microarray studies that is part of the Plant Expression Database [PLEXdb] [16]. Currently, the expression datasets derived from the GeneChip^® ^*Vitis vinifera *(Grape) Genome Array ver. 1.0 (Affymetrix^® ^Inc., Santa Clara, CA) can be deposited here.

Other online specialized databases contain information on *Vitis *ESTs, such as the DFCI grape gene index database that store information from *V. vinifera *ESTs (191,616 in total) including information on the tentative consensus sequences, their BLAST and Gene Ontology details [17]. Also, functional tools are available that allow comparison of EST expression profiles from different libraries, and information on alternate spiced forms, among others. Similarly, EST sequence information along with their microarray probe information can be obtained from the Plant genome database PlantGDB, where about 210,000 *V. vinifera *ESTs are stored [18]. Information on the *V. vinifera *ESTs in different metabolic pathways can be obtained from the KEGG website [19]. A more up-to-date data on the clustered EST sequences from different *Vitis *species with large EST collections can be obtained from the TIGR plant transcript assemblies database [20]. The *V. aestivalis *var. Norton EST database is available at Missouri State Univ.-Mountain Grove and also includes a defense gene database called GREED [21].

VitisExpDB was developed to curate and permit easy access to all the available grape EST sources and integrate with the microarray data, especially data from custom arrays from non-*vinifera *varieties and species, which are a known source of biotic and abiotic stress resistance germplasm. Putative homologs have been identified across different *Vitis *cultivars and species and with the model plant *Arabidopsis*. Several search and retrieval forms along with online bioinformatics tools were developed to create a comprehensive data warehouse for *Vitis *genomics research. The database will be updated every six months with available new data sets.

## Construction and Content

### Database architecture

**Figure 1:**
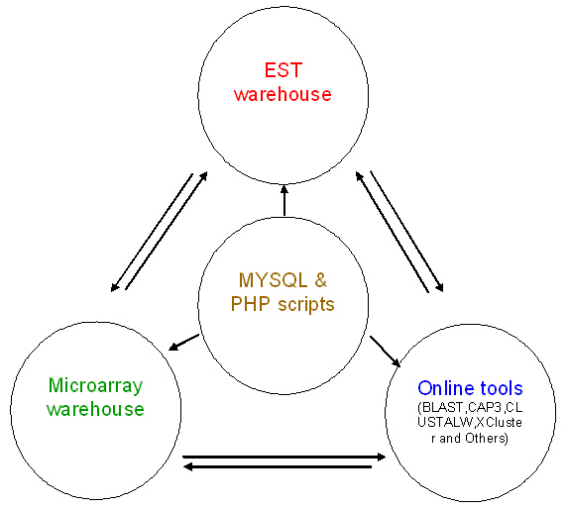
**Overview of the VitisExpDB database**. The three main components are interconnected with sufficient cross-links. Results are generated dynamically with data retrieved from the MYSQL database using the customized PHP scripts.

The server uses Red Hat Enterprise Linux 4 RPM (x86). The relational database was developed using MySQL 4.0 as the back end. The website is powered by an Apache server. HTML- and PHP-based web interfaces have been developed which dynamically execute the MYSQL queries and also run different dependent softwares (Figure 1). Software packages, ADO database and BioPHP, have been used to interact with the MYSQL database.

### Data curation

#### ESTs

EST sets were down loaded from NCBI data banks (UniGene for *V. vinifera*, dbEST for the rest of the *Vitis *species, Table 1) and searched using the BLASTX algorithm against the entire 'nr' protein database using the NCBI BLAST service. These results were reconfirmed by repeating the similarity search using the Personal BLAST Navigator (PLAN) software server [22]. In both the cases, an identity cut-off E value of 10^-4 ^was used. The Gene Ontology terms [23] were generated using the High Throughput Gene Ontology Functional Annotation (Ht-Go-Fat) toolkit [24]. Sequence similarity search was carried out using the default BLAST search parameters and a cut off E value of 10^-4^. The generated GO terms were hyperlinked to their definitions and ontologies in the latest release of the "Gene Ontology, OBO v1.2", downloaded from the GO website [25]. The database also lists similar gene sequences among different species of *Vitis *that were identified using our recently developed nWayComp tool [26]. For this, ESTs from *V. vinifera*, *V. shuttleworthii*, *V. arizonica*, *V. aestivalis*, *V. riparia *were subjected to reciprocal BLAST searches using the BLASTN program with an expectation score cutoff of E-010. Potential applications of identifying such putative homologs across different *Vitis *species include deducing presumptive function of the cloned ESTs, and cloning of ESTs from other varieties based on primers designed from conserved regions. Similarly, such putative homologs can be used to develop SSR and SNP markers for varietal identification and construction of genetic maps for marker assisted breeding. For the *V. vinifera *dataset, the latest expression profiles downloaded from the UniGene built #21 have been wrapped up with the PHP scripts to dynamically generate digital EST expression profiles across nine different tissue types.

#### Microarray datasets

We have designed a *V. vinifera *and *non-vinifera *EST-enriched custom high density microarray gene chip with a total of 20,020 ESTs (1,947 from the SSH libraries, 40 from the cDNA-AFLP experiments, 10,014 from *V. vinifera*, 5,470 from *V*. *shuttleworthii*, 1,219 from *V. aestivalis*, 780 from *V. rupestris *× *V. arizonica *and 588 from *V. riparia*). The database includes analyzed global microarray expression profiles generated in hosts infected with the plant pathogenic bacterium *X. fastidiosa*, which causes Pierce's disease in grapevines. The data were generated from 36 hybridization experiments from three time points: early (1 week), mid (6 weeks) and late (10 weeks) stages of disease development from both infected and non-infected tissues of stem and leaf from resistant and susceptible genotypes. In addition, DNA sequence information on each of the EST sequences on the custom microarrays along with the spotted probe and annotation details is accessible. Further, the generated expression profiles have been mapped onto 25 metabolic pathways using the TAIR's Pathway Tools Omics Viewer [27]. For this, the normalized and fold change calculated expression values for the *Vitis *and *X. fastidiosa *interactions experiments were mapped on to these pathways. Putative homologs *Arabidopsis *gene IDs of the *Vitis *ESTs on the microarray chip were used for this purpose. Details of the microarray experiments can be viewed at the website [27]. Two other published custom microarray datasets [11,12] have also been added to the database. Data will be updated every six months.

## Utility and Discussion

### Web interface

On the main search page, there are two side panels with the panel on the left listing hyperlinks to the different search pages and online tools. The three main components, ESTs, microarrays and the bioinformatics tools, are listed here. The panel on the right lists hyperlinks to the Web Pages that describe the contents of the database. Information, such as experimental set up, data analysis and other relevant text, is provided in these pages. A number of useful query interfaces for data mining, analysis and visualization have been developed. This includes simple and advance search forms that facilitate either single query or multiple query search options for both EST and microarrays components.

**Figure 2:**
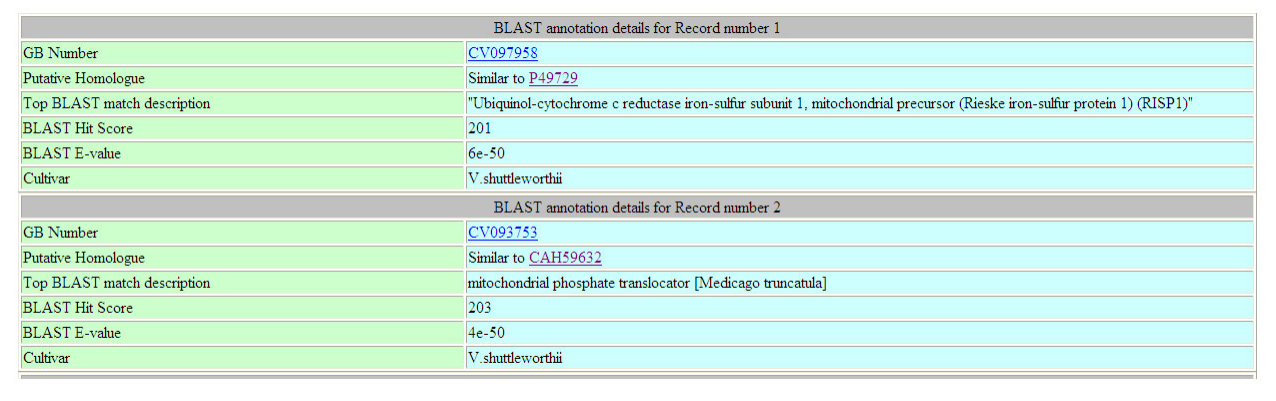
**Example result pages of an EST database query**. This figure shows the upper quartile of the composite image of a page displaying BLAST search results for query term "mitochondria". For full image please see Additional file 2.

The EST component of the database can be searched using GB number, GI number, Gene Ontology ID, enzyme number or putative function as a key word. Other additional parameters, such as selecting a particular species/variety from the dropdown list, setting a cut-off E value and a cut-off BLAST score, can be included to build a stringent query. A typical results page displays ESTs matching the query, individual EST sequence, its description, EC number, and its Gene Ontology classification, (see Figure 2 A-B and see Additional file 3). Extensive hyperlinks have been included in the result pages that will link the result terms to other tabular data in the database and to external databases such as NCBI and AmiGO database (see Figures 2 C and D in Additional file 2). Result pages include both tables and graphs that are dynamically generated by PHP scripts. For *V. vinifera *ESTs that represent a major portion of the ESTs stored, users can also retrieve the digital expression profile as well as percentage expression indices across 9 different tissues types.

**Figure 3:**
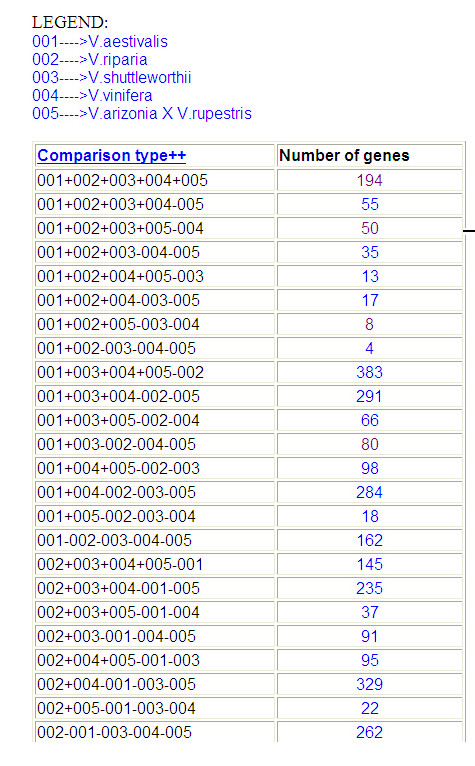
**Example of result pages of putative homologs**. This figure shows the upper quartile of the composite image with the combinations of varieties in the first column and the number of genes for that combination in second column. For full image please see Additional file 3.

A separate Web Page is provided for listing the putative homologs gene sets in the major *Vitis *varieties using nWayComp. This information can be obtained from the 'Get Homologs" link on the left hand side of the main database page. The main result page has a table with two columns with the first column displaying the comparison type and the second column displaying the number of putative homologs gene sets for that combination (Figure 3A, and see Additional file 4 for the full image). Numbers in the second column are hyperlinked to open HTML analysis files that show a table that has all the sets of putative homologs genes for that combination, Figure 3B (see Additional file 3). The first column of this page displays a sorted list of putative homologs gene sets one per *Vitis *species or variety in the ascending order of their sequence similarity. The second column of this table has the standard deviation of sequence identities among putative homologs genes. For each set of putative homologs genes, columns three to six will have icons that are a hyperlinked to the files that will display an identity table showing the percentage of sequence identity among putative homologs genes, column 3; Figure 3C (see Additional file 3), a phylogenetic tree, column 4; Figure 3D (see Additional file 3), common primers used for PCR cloning that are sorted in the descending order of the amplicon size, column 5; Figure 3E (see Additional file 3), and the gene sequences in FASTA forma, column 6; Figure 3F (see Additional file 3).

**Figure 4:**
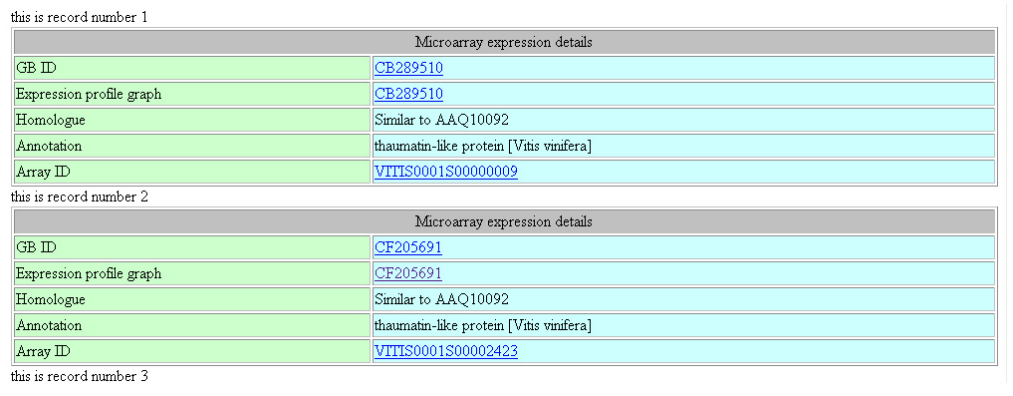
**Example result pages of a Microarray database query**. This figure shows the upper quartile of the composite image of a truncated page displaying annotation (BLAST top match description) search results for query term "thaumatin". For full image please see Additional file 4.

To query the microarray data, a simple form can be used whereby the user can enter a GB number, an array ID(s) or a putative function (Figure 4A- B and Additional file 4). Alternately, an advanced search form is designed whereby the user can build stringent Boolean searches, such as a cut-off expression value or select a particular stage of the experiment, tissue or genotype, or based on *Arabidopsis *genes, for data retrieval. A typical microarray search result will include sequence, annotation and probe details along with a dynamically generated postscript image of the sequence and annotation details along with expression results in the form of a graph, Figure 4C (see Additional file 4). On the advanced search page, an additional form is provided that can be used to retrieve information on microarray probes, Figure 4D (see Additional file 4).

Under the microarray warehouse, a separate HTML page has been designed that has hyperlinked icons to various metabolic pathways. There are 25 different pathways for each of the 12 microarray experiments studied, created as HTML pages from images generated by mapping the differentially regulated *Vitis *ESTs as described under the sub section Microarray datasets. A separate Web Page lists different pathways where the *Arabidopsis *gene IDs has been linked to the putative homologs ESTs in the VitisExpDB to retrieve further information on interesting Arabidopsis genes. In addition, a microarray data repository that will include all the custom microarray data sets is under development.

### Online tools

**Figure 5:**
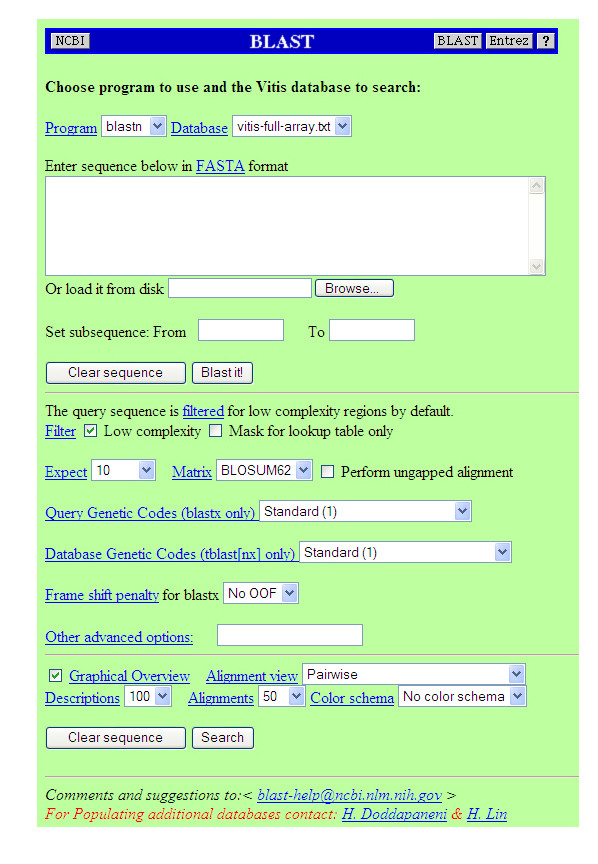
**Screen shot of the online tools and the subsequent result pages**. This figure shows the upper quartile of the composite image (BLAST web interface), for full image please see Additional file 6.

Several online tools, such as BLAST, CLUSTALW, Tandem Repeats Finder (TRF), and Cluster, have been adapted to interface with our database. Others, such as CAP3, and other BioPHP modules (See Additional File 5), can be accessed independently. Annotated *Vitis *databases, such as EST and microarray probe sequence sets, have been added to the BLAST database (*Vitis full sequence BLAST db and Vitis full array BLAST db) *that will help the grape scientific community to quickly and efficiently identify and annotate ESTs. The BLAST tool accepts either single or multiple sequence files in a FASTA format. All the NCBI's BLAST software features have been retained for familiarity of use (Figure 5A, and see Additional file 6 for the full image). The CLUSTALW tool can be used to align up to 20 sequences at a time and the user can input the GB accession for the sequences of interest as a comma separated list, Figure 5B (see Additional file 6). Results files from each run are saved with the user provided file name for subsequent retrieval, Figure 5C (see Additional file 6). A TRF search page has been developed where the user can customize the search for repeats within a sequence as well as try different search options, Figure 5D (see Additional file 6). Output files are saved with the user input options as prefix to avoid overwriting the files, Figure 5E (see Additional file 6). This is significantly different from displaying static pages where pre-run data on tandem repeats is displayed as in most of the EST databases, thereby missing the option of applying different search parameters on a single EST sequence. For analysis of functionally related genes based on their microarray expression profiles, the latest Linux version of the XCluster software was configured [28]. The customized result files from the XCluster can be downloaded onto local hard drives and stored for future use. An EST sequence submission form has been developed where users can submit their sequence directly to the database. A conformation e-mail will be generated alerting the submitter of a successful submission. The added sequences will be annotated monthly, after carrying out quality control analysis, such as vector and adapter contamination, and will be added to the main database for later pubic retrieval. A link to the Web Page has been provided on the left-hand side at the bottom of the main Web Page that lists all the major and interesting *Vitis *EST and genomic resource Web Pages.

In addition to having the most current EST data pool with annotation and Gene Ontology curation, VitisExpDB also has other unique features, such as information on putative homologs from different *Vitis *species, information on their *Arabidopsis *putative homologs, integration of microarray and EST databases, mapping of transcriptional responses on to metabolic pathways and several data analysis tools.

## Conclusion

The VitisExpDB database is a valuable resource for broad applications to *Vitis *genetics and breeding, genomics, proteomics and genome annotation. Future expansion plans of the database include cataloging splice variants from the ESTs, identifying full-length ESTs based on tentative consensus sequences that are backed up by the genomic data and generation of genomic landscape maps of gene expression. Development of cross reference tools for users to compare data between Affymetrix gene chip array and other custom arrays is also planned as a part of future database expansion. The VitisExpDB database will be updated frequently as and when more information becomes available.

## Availability and requirements

The database is open and freely available [29].

Project name: VitisExpDB database;

Project home page: ;

Operating system(s): Platform independent;

Programming language: Perl, HTML, MySQL and PHP;

License: GNU.

## Authors' contributions

HD carried out microarray experiments, designed the database, and developed the web interface and data analysis tools. JY developed the NWayComp software and participated in the initial development of the database. HL and AW helped with the development of similarity search parameters and HL and EC helped with microarray data interpretation. EC secured funding from the California Citrus Research Board. HL, AW and EC coordinated the project. All the authors read and approved the final manuscript.

## Supplementary Material

Additional file 1 Genetic-background of the ESTs curated. Document with details of the genetic background of the grape vines from which ESTs were generated. Click here for file

Additional file 2 Example result pages of a Microarray database query. (A) Screenshot of a truncated page displaying annotation (BLAST top match description) search results for query term "thaumatin". (B) View of a page displaying the FASTA sequences and cultivar details. Further information to an external link to the NCBI's GenBank is provided. (C) View of a page displaying the results of the expression profile in the form of a graph which is generated dynamically, displaying the stage, expression value and the percentage of expression across the 12 data points. Values in the while boxes are fold-change differences for treatment over controls. Percentages given at the top of the bars were calculated by dividing the fold change value for that stage over the fold change across all the 12 stages. (D) View of a page displaying the microarray probe information. Click here for file

Additional file 3 Example result pages of an EST database query. (A) Screenshot of a truncated page displaying BLAST search results for query term "mitochondria". BLAST matches with a E-value of greater than 1e^-6 ^have been described as "*Weakly similar" *and less than that as *"similar to" *(B) Hyperlinked Gene Ontology terms and Enzyme details (C) Description of the related GO terms with hyperlink to the Gen Ontology tree view page for that term at AmiGO database (D) Tree view image of the GO term "GO:0003954". Click here for file

Additional file 4 Example of result pages of putative homologs. (A) View of a page showing the combinations of varieties in the first column and the number of genes for that combination in column two; (B) Putative homologs gene sets in that comparison type, with one set per row in the first column; (C) Percent identity for that putative homologs set; (D) Unrooted neighbor-joining phylogenetic tree constructed using Phylip program with default settings showing their relationship; (E) Truncated list of common primers for that set; and (F) Truncated DNA sequences in FASTA format. Click here for file

Additional file 5 Description of the bioinformatics tools. File has brief description of the usage of bioinformatics tools available. Click here for file

Additional file 6 Screen shot of the online tools and the subsequent result pages. (A) BLAST web interface (B) CLUSTALW web interface (C) results page of the CLUSTALW search with links to the subsequent data files. Users can save these files on to their local hard drives for further analysis and interpretation. (D) TRF web interface (E) summary result page of the tandem repeats search with links to the result pages. Click here for file
