# Supplementary material for: VitisExpDB: A database resource for grape functional genomics
Source: BMC Plant Biol. 2008 Feb 28;8:23. doi: 10.1186/1471-2229-8-23 (PMC2359749; doi:10.1186/1471-2229-8-23)
Supplement: Additional file 1 — Genetic-background of the ESTs curated. Document with details of the genetic background of the grape vines from which ESTs were generated. [file 1471-2229-8-23-S1.doc]

***Genetic background of the ESTs curated***

*Vitis vinifera* L., the European wine grape is the most important cultivated grape cultivar. This species accounts for over 90% of world’s grape production. Most of the production is used to make wine, but it is also the primary species used for table (fresh eating) and raisin grape production. There are at least 5000 cultivars of *V. vinifera* grown worldwide, of which 100 make up the vast majority of production. The most popular white wine cultivar is ‘Chardonnay', and major red wine cultivars include ‘Cabernet Sauvignon', ‘Merlot', and ‘Pinot Noir'. ‘Thompson Seedless' is the most common cultivar used for table grapes and raisins because it is seedless and productive.  *Vitis shuttleworthii* is a North American grape variety native to central and southern Florida. *Vitis shuttleworthii* has relatively large berries and high levels of resistance to Pierce's disease and a host of damaging foliar fungal diseases, as well as moderate resistance to dagger nematodes. It has been used to produce fruiting hybrids for the southeastern US in the past (Fennell). *Vitis aestivalis* is found across the eastern US from Texas to Florida and north to New England. This species has good fruit quality, large berries and good resistance to foliar diseases such as powdery and downy mildew. *Vitis riparia*, also commonly known as River Bank Grape or Frost Grape, is a Native American climbing or trailing vine, widely distributed in North America. The foliage is typically resistant to mildew and black rot, and the roots are resistant to phylloxera. It is has been used extensively in grape breeding programs to transfer cold hardy and disease resistant genes to domesticated grapes. The hybrid, *V. rupestris x V. arizonica* represents siblings of mapping population segregating for Pierces’s disease resistance (1).

1. Krivanek, A.F., Riaz, S., Walker, M.A.(2006) **Identification and molecular mapping of PdR1, a primary resistance gene to Pierce's disease in *Vitis***,
   *Theor. Appl. Genet.,* **112**, 1125-1131.
