# Supplementary figures and images for: VitisExpDB: A database resource for grape functional genomics
Source: BMC Plant Biol. 2008 Feb 28;8:23. doi: 10.1186/1471-2229-8-23 (PMC2359749; doi:10.1186/1471-2229-8-23)

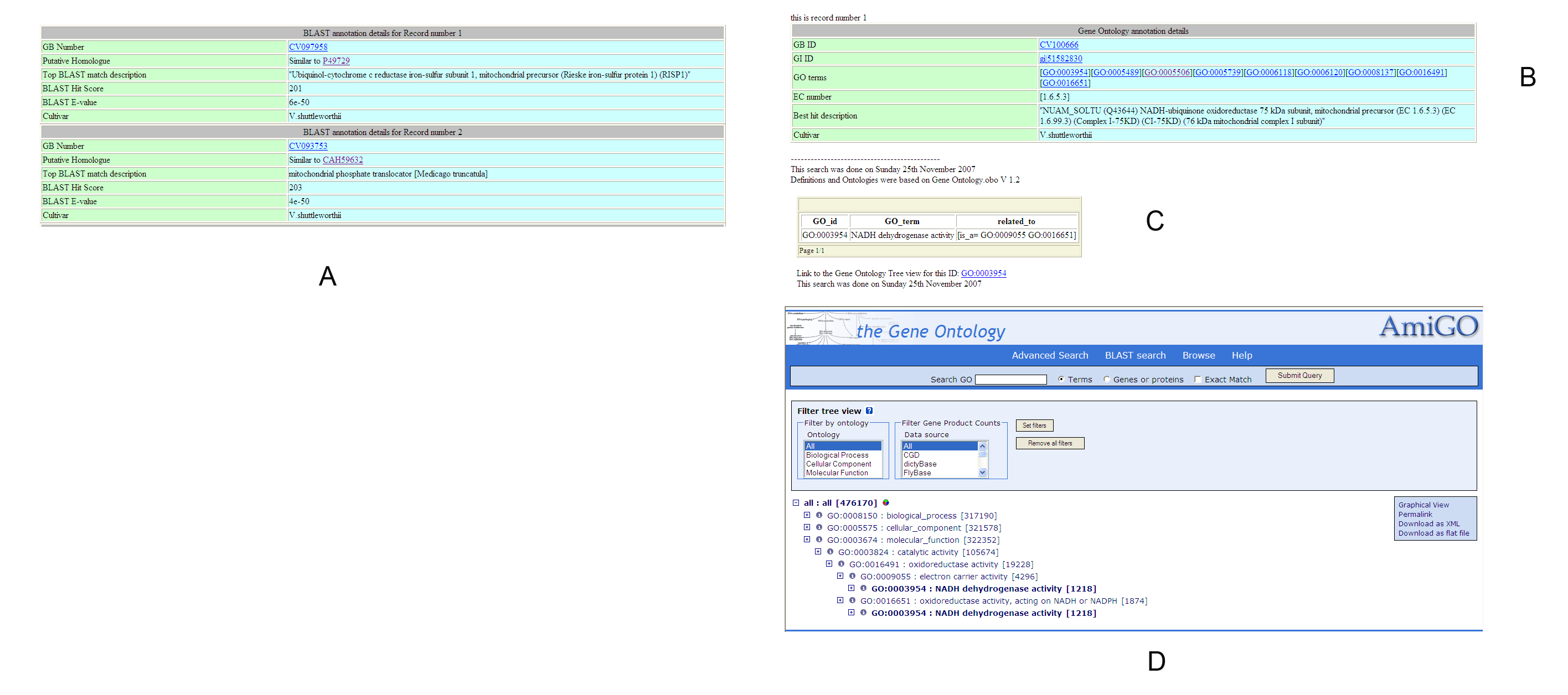

Supplement: Additional file 2 — Example result pages of a Microarray database query. (A) Screenshot of a truncated page displaying annotation (BLAST top match description) search results for query term "thaumatin". (B) View of a page displaying the FASTA sequences and cultivar details. Further information to an external link to the NCBI's GenBank is provided. (C) View of a page displaying the results of the expression profile in the form of a graph which is generated dynamically, displaying the stage, expression value and the percentage of expression across the 12 data points. Values in the while boxes are fold-change differences for treatment over controls. Percentages given at the top of the bars were calculated by dividing the fold change value for that stage over the fold change across all the 12 stages. (D) View of a page displaying the microarray probe information. [file 1471-2229-8-23-S2.jpeg]

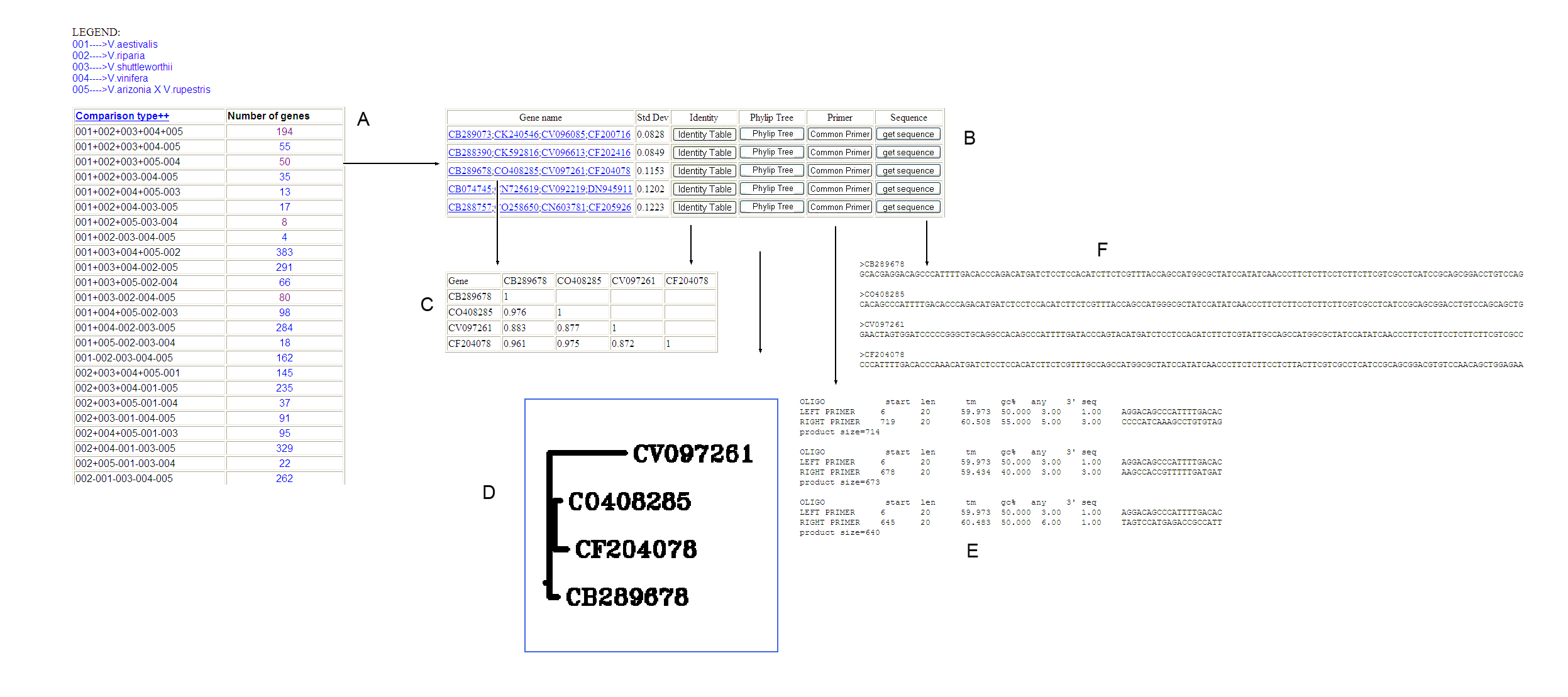

Supplement: Additional file 3 — Example result pages of an EST database query. (A) Screenshot of a truncated page displaying BLAST search results for query term "mitochondria". BLAST matches with a E-value of greater than 1e-6 have been described as "Weakly similar" and less than that as "similar to" (B) Hyperlinked Gene Ontology terms and Enzyme details (C) Description of the related GO terms with hyperlink to the Gen Ontology tree view page for that term at AmiGO database (D) Tree view image of the GO term "GO:0003954". [file 1471-2229-8-23-S3.jpeg]

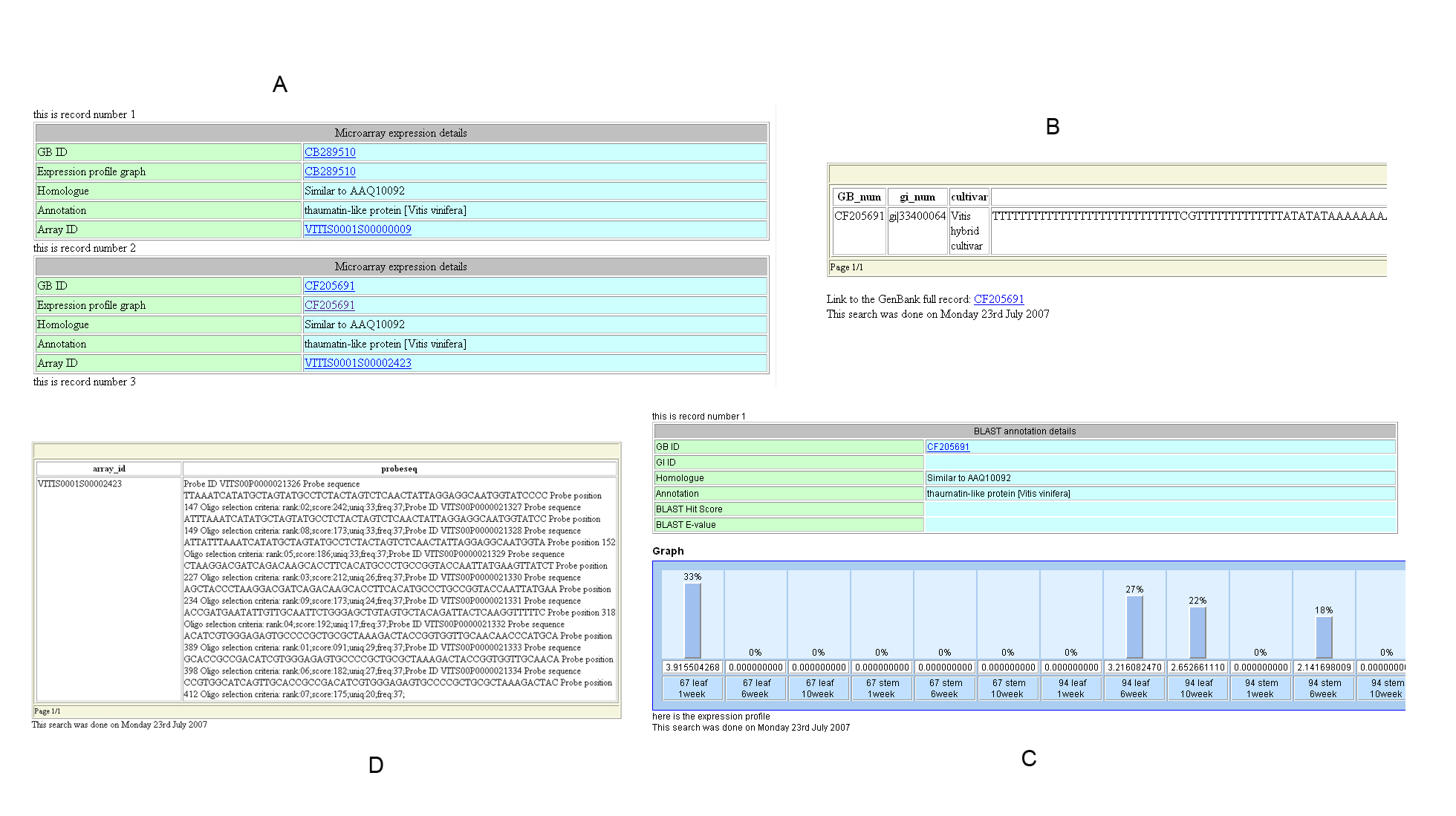

Supplement: Additional file 4 — Example of result pages of putative homologs. (A) View of a page showing the combinations of varieties in the first column and the number of genes for that combination in column two; (B) Putative homologs gene sets in that comparison type, with one set per row in the first column; (C) Percent identity for that putative homologs set; (D) Unrooted neighbor-joining phylogenetic tree constructed using Phylip program with default settings showing their relationship; (E) Truncated list of common primers for that set; and (F) Truncated DNA sequences in FASTA format. [file 1471-2229-8-23-S4.jpeg]

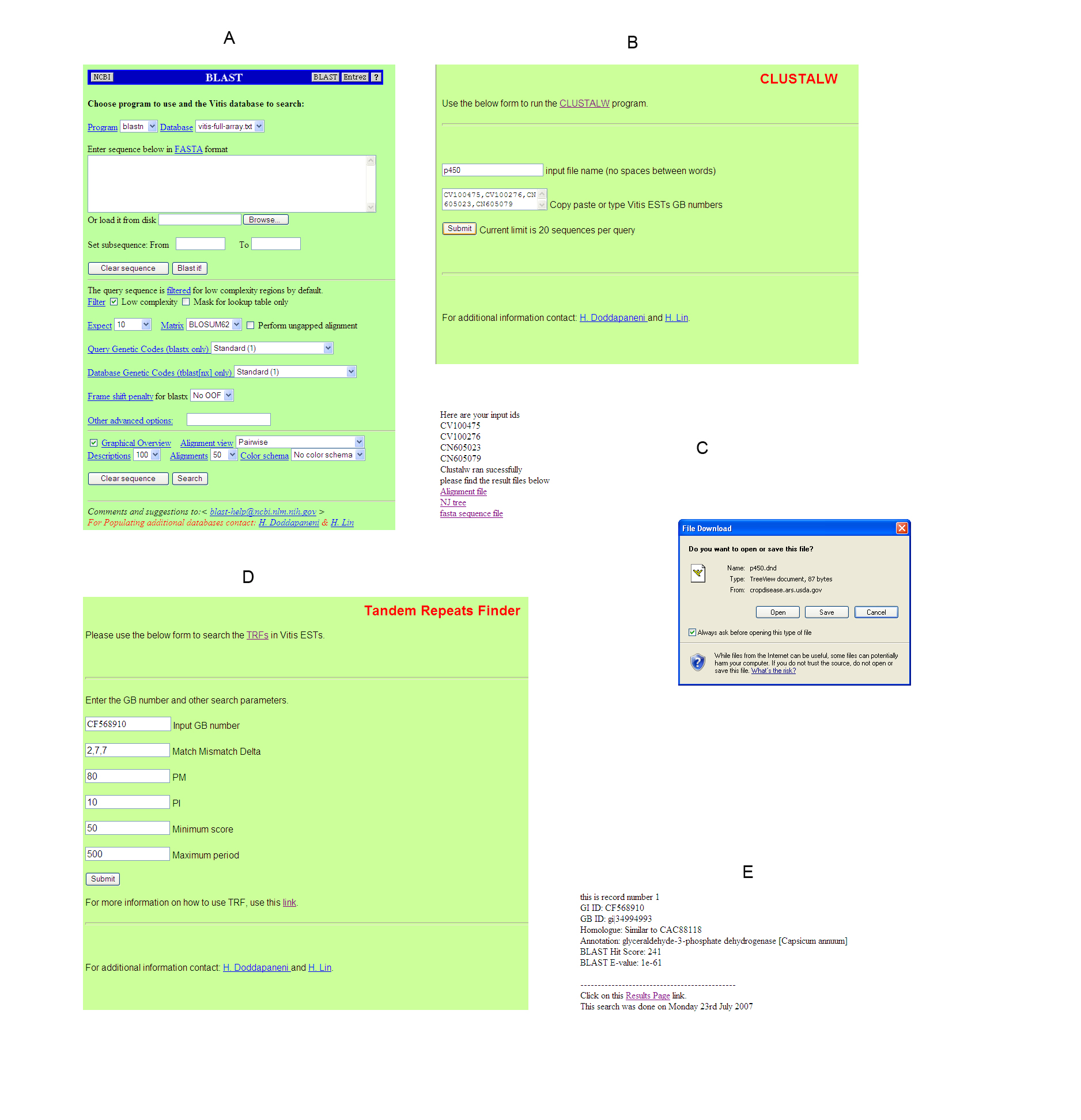

Supplement: Additional file 6 — Screen shot of the online tools and the subsequent result pages. (A) BLAST web interface (B) CLUSTALW web interface (C) results page of the CLUSTALW search with links to the subsequent data files. Users can save these files on to their local hard drives for further analysis and interpretation. (D) TRF web interface (E) summary result page of the tandem repeats search with links to the result pages. [file 1471-2229-8-23-S6.jpeg]
