# Supplementary material for: VitisExpDB: A database resource for grape functional genomics
Source: BMC Plant Biol. 2008 Feb 28;8:23. doi: 10.1186/1471-2229-8-23 (PMC2359749; doi:10.1186/1471-2229-8-23)
Supplement: Additional file 5 — Description of the bioinformatics tools. File has brief description of the usage of bioinformatics tools available. [file 1471-2229-8-23-S5.doc]

**A brief description of the other online tools available at the VitisExpDB.**

**CAP3 tool**

CAP3 is a DNA sequence assembly program that computes overlaps between reads, constructs multiple sequence alignments and generates consensus sequences. The program has the capability to use forward-reverse sequence constraints to correct assembly errors and link contigs. User is required to enter a file name and DNA sequences in fasta format. A total of six result files including the contig and singlet files are generated that can be saved on to the local disc. For now, no size constraint has been imposed on the input sequence.

**BioPHP tools**

**DNA to protein translation**

This program translates the input DNA sequence into protein sequence. Translation can be carried out in 1, 3 or all the six frames. DNA sequence may be added as shown in the example input or in any other format (number, spaces and line feeds are removed). Also, there are options to remove extra spaces in the input sequence, reverse complement and reverse the sequence. Program can also align the translated amino acid sequence to the input DNA sequence. For translation, a default code can be used or users have the option of choosing the appropriate genetic code.

**DNA sequence manipulation/properties**

This program has multiple functions. Using this tool, a variety of routine DNA manipulation tasks can be performed such as, removing the non-coding characters in the sequence, reversing the sequence, reverse complement, to show the complementary strand sequence, and to convert DNA into RNA sequence. In addition, GC content and nucleotide composition of the entire sequence or a subset of the sequence can be determined.

**Frequency of nucleotides and oligonucleotides**

This tool will count the number of occurrences of a oligonucleotide with length between 1 and 8 within the input sequence. Frequency can be calculated on one or both the strands. 10 Kb is the limit on the input sequence size.

**PCR amplification**

This program simulates the PCR amplification. Out put includes the forward and reverse primer sequence, size of the amplicon and the sequence of the amplicon.

**Melting Temperature (Tm) Calculation**

This tool calculates the melting temperature (Tm) of an oligonucleotide. The program assumes that there is 50 nM concentration of primer, 50 mM Na^+^, and solvent is at a pH of 7.0.

**Microarray Data Analysis** for two color experiments

This program uses adaptative quantification method to analyze microarray results. For each spot, values are calculated based on the formula (value-background) *100/sum (value-background)) for both red and green channels. When there are several replicas for ch1/ch2 and ch2/ch1, median and its base 10 logarithm is computed. In the results table, medians and logs are presented. Table has a color code with red color representing over-expression and blue color, repression.

**Protein to DNA reverse translation**

This program outputs the DNA sequence based on an input protein sequence. Reverse translation is carried out on all the six frames. This program is useful in designing degenerate oligonucleotide primers. Users have the potion of choosing several genetic codes.

**Restriction enzyme digestion of DNA**

This program generates restriction digestion profile of the input DNA sequences with selected or all the available endonucleases. The program allows restriction of one or more sequences, and also the comparison of restriction patterns. Recognition sites for all the commercially available restriction enzymes are included in the program.
